# No Trace of *Blastocystis* Parasite in Bats Caught in the Western and Southwestern Regions of Iran

**DOI:** 10.1002/vms3.71242

**Published:** 2026-09-29

**Authors:** Mohammad Amin Mosayebi, Ali Pouryousef, Mostafa Omidian, Saeed Shahabi, Bahador Sarkari

**Affiliations:** ^1^ Department of Parasitology and Mycology School of Medicine Shiraz University of Medical Sciences Shiraz Iran; ^2^ Leishmaniasis Research Center Sabzevar University of Medical Sciences Sabzevar Iran; ^3^ Department of Biology and Control of Disease Vectors School of Health Shiraz University of Medical Sciences Shiraz Iran

**Keywords:** chiroptera, DNA extraction, faecal samples, molecular detection, zoonotic pathogens

## Abstract

**Background:**

*Blastocystis* is a common parasite found in the human intestine, causing symptoms such as abdominal pain, bloating, and diarrhoea. Bats are potential hosts for this parasite, but little is known about their infection with *Blastocystis*.

**Objective:**

Given the diversity of bat species in Iran, this study aimed to investigate *Blastocystis* infection in bats caught in the southwestern and western regions of the country.

**Methods:**

Faecal samples were collected from 115 bats from five caves located in Fars, Kurdistan, and Kermanshah provinces. DNA was extracted, using the phenol‐chloroform method, and PCR was performed to identify the *Blastocystis* 18S rRNA gene. Information on bat species was analysed descriptively.

**Results:**

The four main bat species caught were *Miniopterus pallidus* (40%, n = 46), *Myotis blythii* (40%, n = 46), *Rhinopoma microphyllum* (12.18%, n = 14), and *Rhinolophus euryale* (7.82%, n = 9). However, none of the 115 stool samples examined showed evidence of *Blastocystis* infection by the molecular PCR method.

**Conclusion:**

This study found no evidence of *Blastocystis* infection in the bat species examined. The unique lifestyle and diet of bats, such as living on cave walls and roofs, may explain this absence. Further studies are needed to clarify the role of bats in the epidemiology of *Blastocystis* in other regions of Iran.

## Graphical abstract

Despite screening 115 bats across four dominant species (Miniopterus pallidus, Myotis blythii, Rhinopoma microphyllum, and Rhinolophus euryale) from five caves in western and southwestern Iran using PCR, no evidence of *Blastocystis* infection was found, suggesting these bats are not current reservoirs for this parasite in this region.

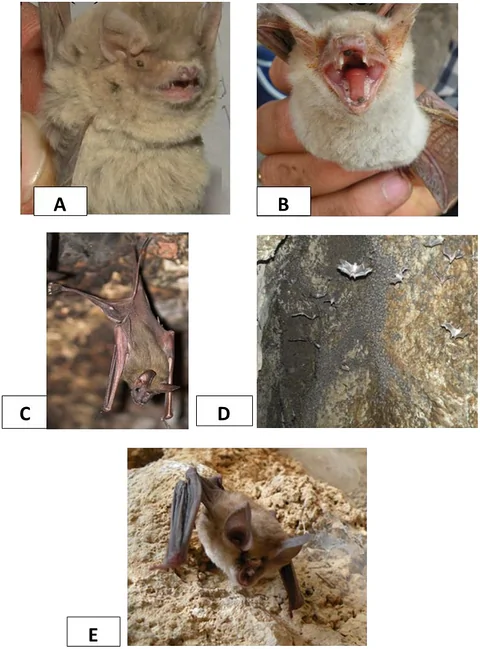

## 1 Introduction

*Blastocystis* is a unicellular, anaerobic protozoan parasite that inhabits the gastrointestinal tract of humans and a wide range of animals, exhibiting significant genetic diversity and potential zoonotic importance (Tan 2008). The main route of transmission of *Blastocystis* is through water and food contaminated with pathogenic cysts of this parasite (Clark et al. 2013). In humans, *Blastocystis* infection is associated with a spectrum of clinical manifestations, ranging from asymptomatic carriage to gastrointestinal symptoms such as acute or chronic diarrhoea, abdominal pain, bloating, and irritable bowel syndrome‐like disorders, though its pathogenicity remains debated (Tan 2008).

Globally, *Blastocystis* displays extensive genetic diversity, with over 40 subtypes (STs) now identified based on small subunit ribosomal RNA (SSU rRNA) gene sequence analysis. Subtypes ST1–ST4, ST6–ST9, and ST12 are commonly reported in humans, with ST3 frequently predominant in many populations, while higher‐numbered STs (e.g., ST10–ST17 and beyond) are predominantly animal‐specific, though some overlap suggests zoonotic potential (Stensvold and Clark 2016). Meta‐analyses have estimated pooled prevalences in various hosts: approximately 18%–21% in rodents worldwide, 29% in birds, and variable rates in other wildlife and livestock, highlighting the role of animals as reservoirs (Barati et al. 2022; Farzam et al. 2025; Zanetti et al. 2020). In wildlife, studies have documented *Blastocystis* in diverse mammals and birds, with subtypes often shared across hosts, underscoring epidemiological links to human infections.

In Iran, *Blastocystis* is prevalent in humans and domestic animals, with pooled estimates from systematic reviews indicating infection rates around 3%–10% in human populations depending on region and detection method, and higher in certain animal groups such as ruminants (e.g., up to 14% in southwestern provinces) and camels (Heydarian et al. 2024). Common subtypes in Iranian humans and livestock include ST1–ST3 (often zoonotic) and animal‐associated ST5, ST10, and ST14, with evidence of shared genotypes between hosts reinforcing zoonotic transmission risks (Maleki et al. 2022; Mohammadi et al. 2025). National studies on wildlife, including birds (e.g., crows and pigeons in Tehran showing ST13 and ST14) and cattle in western provinces (e.g., ST5 predominant), further illustrate regional diversity and potential reservoirs (Asghari et al. 2019; Sharifi et al. 2020).

Bats (Order Chiroptera) represent the second most speciose mammalian group after rodents, with high ecological diversity and known roles as reservoirs for numerous zoonotic pathogens, including viruses, bacteria, and protozoa. Iran harbours substantial bat biodiversity, with at least 51 species documented across varied habitats (Benda et al. 2012). Despite this, parasitological research on Iranian bats has largely focused on ecology, taxonomy, and molecular phylogeny, with limited attention to protozoan parasites like *Blastocystis* (Martinez‐Hernandez et al. 2020; Shahabi et al. 2017a, Shahabi et al. 2017b; Shahabi et al. 2019). Internationally, studies on *Blastocystis* in bats remain scarce and often report low or absent infection. For instance, in Mexico, only one bat sample was positive among 124 wild animal faeces examined (Martinez‐Hernandez et al. 2020); while in China, no Blastocystis was detected in 10 fruit bat faecal samples despite high *Cryptosporidium* prevalence (Zhao et al. 2017). Recent global characterisations have occasionally identified novel or rare subtypes in bats (e.g., in studies expanding ST designations), but overall prevalence appears minimal compared to rodents, birds, or livestock (Betts et al. 2018; Maloney et al. 2022).

Given the widespread distribution of *Blastocystis* in humans and animals, its zoonotic potential, and the critical epidemiological role of wildlife reservoirs in transmission cycles, data on bats are notably deficient—particularly in regions like Iran with rich chiropteran diversity and ongoing human‐animal interface pressures. This gap hinders comprehensive understanding of Blastocystis epidemiology, subtype circulation, and potential bat contributions to zoonotic risk. The present study  aimed to investigate the prevalence and molecular subtypes of *Blastocystis* in bat species captured in the western and southwestern regions of Iran, providing baseline data to elucidate whether these mammals serve as reservoirs for this protozoan parasite.

## 2 Materials and Methods

### 2.1 Study Area and Sampling

Samples were collected from 115 bats captured from five caves across Iran in 2021–2022, including three in Fars province (coordinates: Fars Province, Jahrom 28.83°N, 53.33°E, Fars Province, Marvdasht, 30.05°N, 52.73°E and Fars Province, Ghader‐Abad 30.31°N, 53.3°E) and two in Kurdistan (Kurdistan province, Bijar, 35.89°N, 47.58°E) and Kermanshah provinces (Kermanshah Province, Kerend 34.32°N, 46.17°E) (Figure 1). Bats were caught, using hand nets, placed in separate cloth bags, and their faeces were collected after approximately an hour and then the bats were released. A portion of each sample was transferred to 95% ethanol solution and stored at −20°C until use. Bat species were identified morphologically by our trained biologist (Dr. S. Shahabi), using standard field guides and keys (e.g., based on fur colour, ear shape, and forearm length).

**FIGURE 1:**
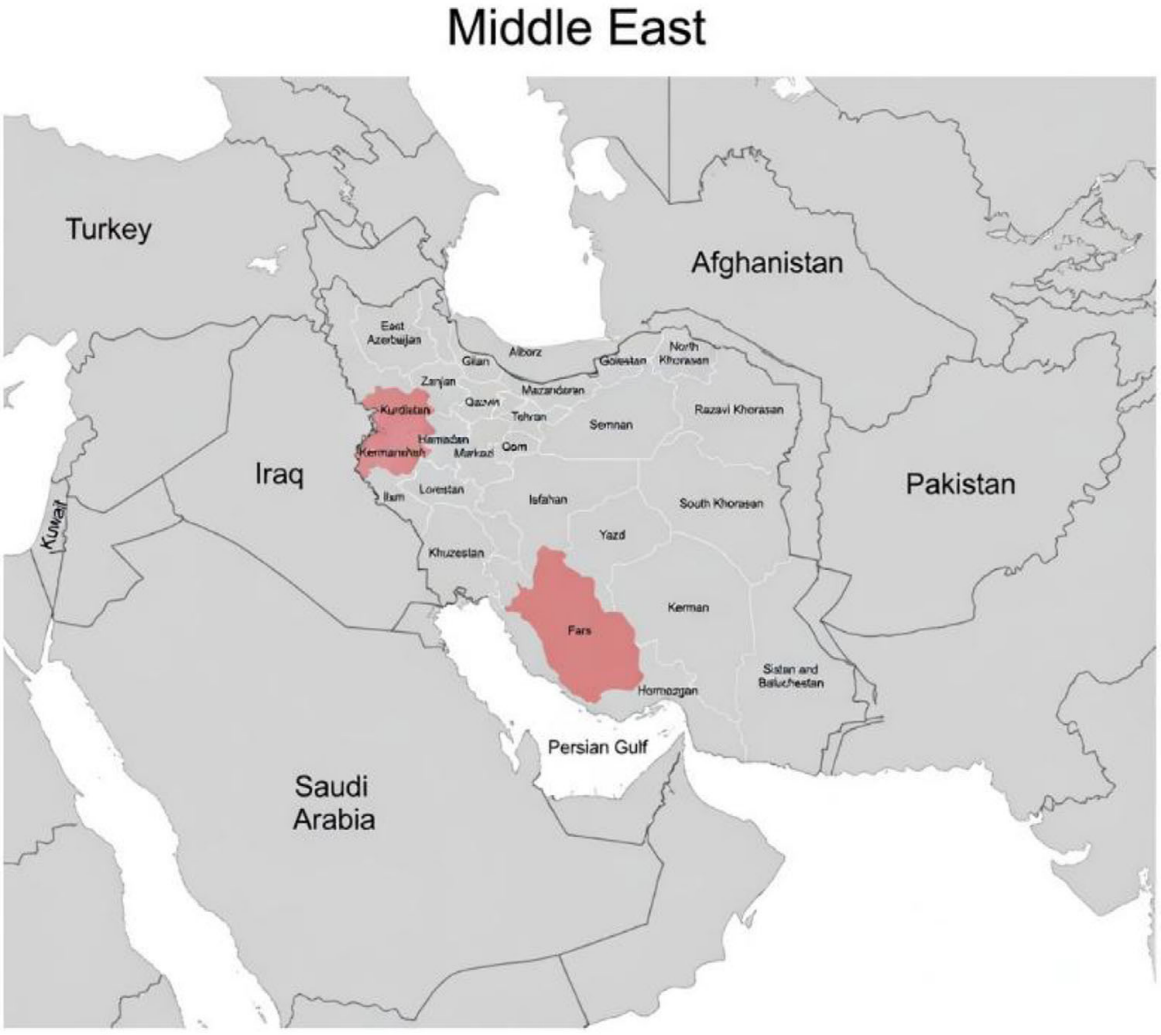
Sampling areas of bats on the map of Iran.

### 2.2 DNA Extraction From the Bats’ Stool Samples

About 100–150 mg of each bat faeces sample was used for DNA extraction, which was performed using the phenol‐chloroform method. The sample was transferred to a 1.5 Eppendorf microtube and then centrifuged for 10 min at 1000 g until the stool sample was completely settled. The supernatant was slowly drained, and the microtube contents were thoroughly mixed with the remaining ethanol. Again, the samples were centrifuged for 10 min. This continued until the liquid on the top of the stool sample became completely clear and bright. Finally, the supernatant was discarded and the samples were ready for DNA extraction. (Sharifi et al. 2020) DNA was extracted from stool samples with a DNA Stool mini kit (YTA, Yekta Tajhiz Azma, Iran), according to the manufacturer's protocol. Extracted DNA was quantified using a NanoDrop spectrophotometer (Thermo Fisher Scientific) to ensure concentrations between 20 and 100 ng/µL and purity ratios (A260/A280) of 1.8–2.0 prior to PCR amplification.

### 2.3 Polymerase Chain Reaction (PCR)

The study utilised PCR to identify the S rRNA gene of *Blastocystis* (Zhao et al. 2017). A specific primer, based on our previous study (Sharifi et al. 2020), was employed to detect *Blastocystis* infection. These RD5 (5′‐ATCTGGTTGATCCTGCCAGT‐3′) and BhRDr (5′‐GAGCTTTTTAACTGCAACAACG‐3′) primers detect a 600 bp fragment of the S rRNA gene of *Blastocystis*. The PCR contents consisted of 5.5 µL of DNA, 7.5 µL of 2 M master mix, 2 µL of primers, and distilled water. The PCR programme included an initial step of 5 min at 95°C, followed by 35 cycles consisting of 30 s at 95°C, 30 s at 55°C, and 2 min at 72°C. The final step was 7 min at 72°C. To enhance the specific bands, if any band was present, a second round of PCR was conducted, using the products and previous primers if the bands were weak in the original PCR. The positive control sample in this study was isolated from a human stool sample in our previous study (Sharifi et al. 2020). This sample was molecularly analysed, and its sequence was determined and registered in GenBank under the accession number MN014071. Electrophoresis was then used to separate DNA fragments, and ethidium bromide dye was utilised for visualisation purposes.

## 3 Results

In this study, a total of 115 faecal samples were collected from bats that were captured in five different caves across three provinces in Iran, namely Fars, Kurdistan and Kermanshah.

The captured bats belonged to five different genera and species (Figure 2). Among the 115 bats, *Miniopterus pallidus* or long‐winged bats were the most common with 46 cases (40%). *Myotis blythii* was ranked second in terms of frequency with 46 cases (40%). *Rhinopoma micophyllum* accounted for 14 cases (12.18%), while *Rhinolophus euryale* had the lowest frequency with only 9 cases (7.82%). Table 1 provides further details on the genus and species of the bats.

**FIGURE 2:**
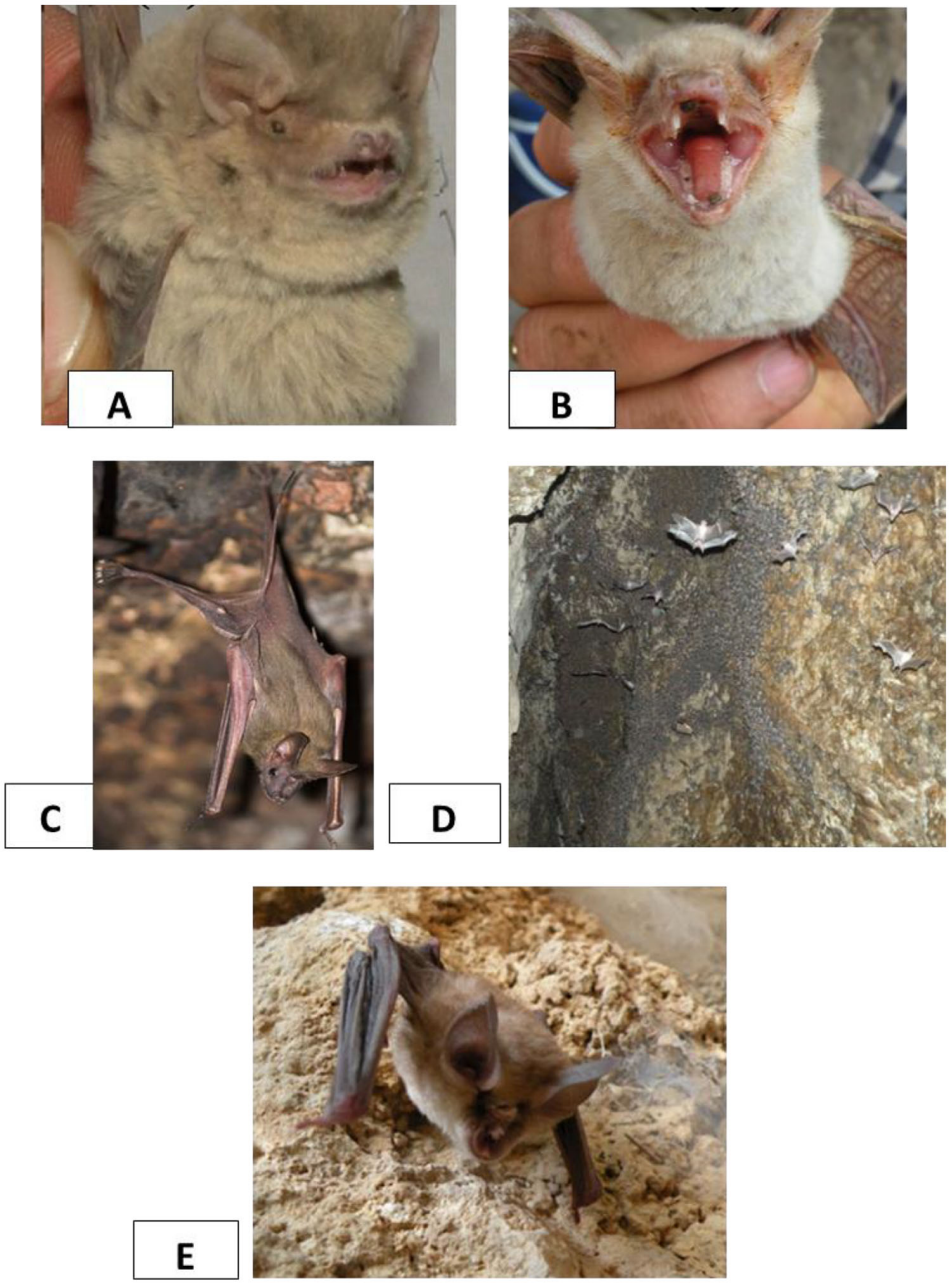
Bats caught in the present study (photo by Dr. Saeed Shahabi). A: *Miniopterus pallidus;* B: *Myotis blythii;* C: *Rhinopoma microphyllum*; D: Large colony of *Rhinopoma microphyllum;* E*: Rhinolophus Euryale*.

**TABLE 1:** The frequency of genus and species of bats caught in three regions (Fars, Kurdistan and Kermanshah provinces) of Iran in the present study.

Row	Genus—species	Common name	Frequency (percent)
1	*Miniopterus pallidus*	long‐winged bat	15 (13.4)
2	*Myotis blythii*	Mouse‐eared bat	2 (1.74)
3	*Miniopterus pallidus*	Long‐winged bat	16 (13.91)
4	*Myotis blythii*	Mouse‐eared bat	22 (19.13)
5	*Miniopterus pallidus*	Long‐winged bat	15 (13.04)
6	*Myotis blythii*	Mouse‐eared bat	12 (10.43)
7	*Myotis blythii*	Mouse‐eared bat	10 (8.69)
8	*Rhinopoma microphyllum*	Mouse‐tailed bat	14 (12.18)
9	*Rhinolophus euryale*	Mediterranean horseshoe bat	9 (7.82)

*Blastocystis* DNA was not detected in any of the 115 examined stool samples of the bats using the molecular PCR method (Figure 3).

**FIGURE 3:**
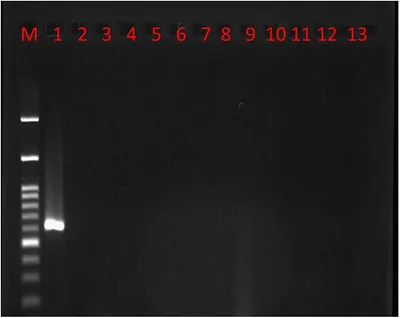
Gel electrophoresis image of PCR product. Lane M: 100–1500 bp molecular marker; Lane 1: *Blastocystis* positive sample (isolated from human); Lane 2: negative control, Lanes 3–13, a few of DNA samples isolated from bat faeces (uncropped gel is submitted as supplementary file).

## 4 Discussion

*Blastocystis* is a parasite that is commonly found in both humans and animals, and it exhibits high genetic diversity. As there are shared subtypes of *Blastocystis* between humans and animals, it is possible for some human infections to be transmitted through animals (Popruk et al. 2021).

The absence of *Blastocystis* DNA in all 115 faecal samples from bats captured in the western and southwestern regions of Iran represents a key finding of the current study. This result aligns with the limited existing literature on Blastocystis in bats (Order Chiroptera), which consistently indicates low‐to‐absent prevalence across diverse geographic regions and bat species.

Globally, *Blastocystis* is a ubiquitous intestinal protozoan with extensive genetic diversity (>40 subtypes identified to date based on SSU rRNA gene analysis) and documented zoonotic potential through shared subtypes between humans and animals (Maloney et al. 2022; Popruk et al. 2021).

While the parasite is prevalent in many mammalian and avian hosts—including rodents, livestock, birds and synanthropic animals—bats appear to be an exception. Previous surveys have reported sporadic or negligible detection: for example, only one positive bat sample among 124 wild animal faeces in Mexico (Martinez‐Hernandez et al. 2020) and no detections in 10 fruit bat faecal samples from Hainan, China, despite concurrent high *Cryptosporidium* prevalence (Zhao et al. 2017). Broader wildlife and zoo‐based studies, including those in Europe, Brazil and other regions, have similarly rarely or never identified Blastocystis in chiropterans, even when screening diverse mammalian groups (Alfellani et al. 2013; Asghari et al. 2021; Sarkari et al. 2016; Sharifi et al. 2020). This pattern suggests potential host specificity barriers or ecological factors limiting *Blastocystis* colonisation in bats.

In Iran, *Blastocystis* is well‐documented in humans (pooled prevalence ∼3%–10% in meta‐analyses, often ST1–ST3) and various domestic/wild animals (e.g., 42.9% in crows, 44.4% in pigeons, 18.8% in dogs, 17.7% in cats, 15.8% in rats and up to 25% in wild boars or cattle in western/southern provinces) (Asghari et al. 2019; Asghari et al. 2020; Sharifi et al. 2020). These findings highlight active circulation and zoonotic risk in the region, particularly in areas overlapping with bat habitats such as Fars province. However, no prior studies have specifically examined *Blastocystis* in Iranian bats, making our investigation the first to address this gap. The complete absence of infection in our samples—from species including *Miniopterus pallidus*, *Myotis blythii*, *Rhinopoma microphyllum* and *Rhinolophus euryale*—reinforces the emerging view that bats may not serve as significant reservoirs for this protozoan.

Several factors may explain this lack of detection. Bats' roosting behaviour in large colonies on cave ceilings minimises direct contact with soil or contaminated ground, a primary environmental source of *Blastocystis* cysts. Their predominantly insectivorous (or frugivorous in some species) diet likely reduces exposure to faecal‐oral transmission routes common in ground‐foraging or omnivorous hosts. Rapid intestinal transit and unique gut physiology in flying mammals may further limit protozoan establishment. These ecological traits contrast with those of rodents or livestock, where higher prevalence is observed.

A key limitation of this study is the exclusive use of SSU rRNA gene‐targeted PCR for detection, without complementary morphological examination or an additional molecular marker. Although molecular methods targeting this gene are the most sensitive and specific available (with reported sensitivities often exceeding 90% compared to <50% for microscopy), the absence of multi‐method confirmation (e.g., microscopy, culture or a secondary gene target) could theoretically overlook very low‐burden or morphologically distinct infections. However, given the consistently negative results across our samples and the documented low‐to‐absent prevalence of *Blastocystis* in bats from other regions, this is unlikely to have impacted our overall conclusion of no detectable infection. Future studies in Iranian or other bat populations could benefit from combining PCR with morphological screening or additional markers to further validate negative findings.

## 5 Conclusion

The lack of *Blastocystis* in examined Iranian bats contributes valuable negative data to the field, suggesting limited zoonotic risk from these mammals in the studied regions. This finding underscores the need for continued surveillance of wildlife reservoirs while highlighting bats' apparent marginal role in *Blastocystis* epidemiology compared to other hosts.

## Author Contributions

**Mohammad Amin Mosayebi**: investigation, methodology, data curation. **Saeed Shahabi**: investigation, methodology, formal analysis, data curation. **Ali Pouryousef**: investigation, writing – original draft, methodology, data curation. **Mostafa Omidian**: investigation, methodology, writing – original draft, formal analysis, data curation. **Bahador Sarkari**: conceptualisation, investigation, funding acquisition, writing – review and editing, validation, formal analysis, supervision.

## Funding

The study was financially supported by the office of the Vice‐Chancellor for Research, Shiraz University of Medical Sciences (grant No. 21585).

## Ethics Statement

Ethics approval of the study was obtained from the ethical committee of Shiraz University of Medical Sciences (SUMS, ref. No. 588.IR.sums.med.rec.1399)

## Data Availability

The datasets used and analysed during the current study are available from the corresponding author upon reasonable request.
